# P-2000. Fast-Tracking Encephalitis Diagnosis: A Molecular Approach to Streptococcus pneumoniae and AMR Gene Detection in CSF Samples

**DOI:** 10.1093/ofid/ofaf695.2164

**Published:** 2026-01-11

**Authors:** Shahreen Rahman, Mohammad Enayet Hossain, Jenifar Quaiyum Ami, Akash Saha, Subyeta Binte Sarwar, Dewan Rahman, Md Zakiul Hassan, Sharmin Sultana, Peter Horby, Piero Olliaro, Sayera Banu, Tahmina Shirin, Joel M Montgomery, Syed Moinuddin Satter, Mohammed Ziaur Rahman

**Affiliations:** icddr,b, Dhaka, Dhaka, Bangladesh; icddr,b (International Centre for Diarrhoeal Disease Research, Bangladesh), Dhaka, Dhaka, Bangladesh; icddr,b, Dhaka, Dhaka, Bangladesh; icddr,b, Dhaka, Dhaka, Bangladesh; icddr,b, Dhaka, Dhaka, Bangladesh; icddr,b, Dhaka, Dhaka, Bangladesh; icddr,b; International Severe Acute Respiratory and Emerging Infection Consortium (ISARIC), Pandemic Sciences Institute, University of Oxford, Oxford, UK, Dhaka, Dhaka, Bangladesh; Institute of Epidemiology, Disease Control and Research (IEDCR), Dhaka, Dhaka, Bangladesh; International Severe Acute Respiratory and Emerging Infection Consortium (ISARIC), Pandemic Sciences Institute, University of Oxford, Oxford, UK, Oxford, England, United Kingdom; International Severe Acute Respiratory and Emerging Infection Consortium (ISARIC), Pandemic Sciences Institute, University of Oxford, Oxford, UK, Oxford, England, United Kingdom; icddr,b (International Centre for Diarrhoeal Disease Research, Bangladesh), Dhaka, Dhaka, Bangladesh; Institute of Epidemiology, Disease Control and Research (IEDCR), Dhaka, Dhaka, Bangladesh; Centers for Disease Control and Prevention (CDC), Atlanta, Georgia; icddr,b (International Centre for Diarrhoeal Disease Research, Bangladesh), Dhaka, Dhaka, Bangladesh; icddr,b (International Centre for Diarrhoeal Disease Research, Bangladesh), Dhaka, Dhaka, Bangladesh

## Abstract

**Background:**

Encephalitis is a significant global cause of morbidity and often causes permanent neurological deficits. It can stem from diverse viral and bacterial infections, with the mortality rate varying by region due to differences in prevalent pathogens. In Bangladesh, the Nipah virus, an endemic zoonotic pathogen in the region, has an alarming case fatality rate exceeding 75% and causes recurrent encephalitis outbreaks. Many encephalitis cases associated with a history of consuming raw date palm sap remain undiagnosed, suggesting that pathogens other than Nipah virus - including *Streptococcus pneumoniae -* may also play important roles. Conventional diagnostic methods for *S. pneumoniae* are time-consuming and costly, and handling samples that may contain potentially high-risk pathogens such as Nipah virus remains challenging in low-resource settings. The recent expansion of molecular biology infrastructure post-COVID-19 offers new opportunities for rapid, non-culture-based diagnostics.

**Figure 01:**
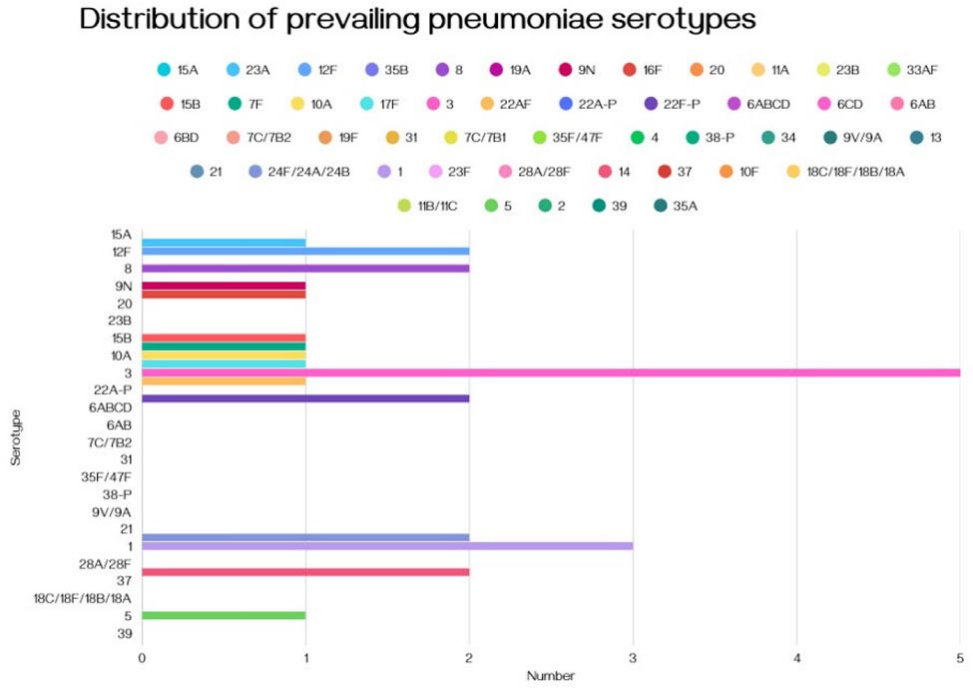
Bar graph showing the distribution of prevailing pneumonia serotypes in the study population

**Figure 02:**
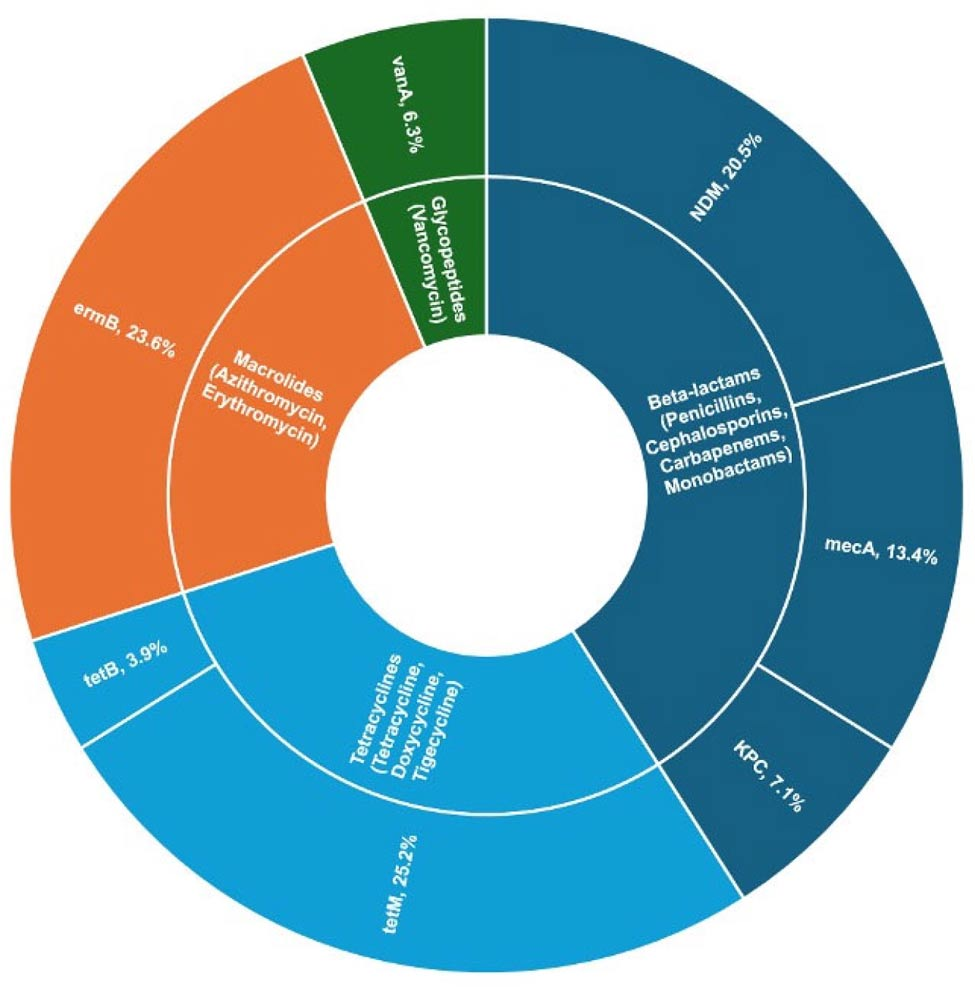
A pie chart depicting the prevalence of antimicrobial-resistant genes in the study population

**Methods:**

We developed a real-time qPCR-based approach for the detection and characterization of viral and bacterial pathogens and antimicrobial resistance (AMR) genes in cerebrospinal fluid (CSF) samples of suspected encephalitic cases. A total of 520 CSF samples were considered for this study, collected between June 2024 and January 2025.

**Results:**

Out of 520 samples, 35 tested positive for*S. pneumoniae through detection of* the *lytA* gene, with 22 serotypes (considering PCV-10, 13, and 23) accurately identified. Additionally, we characterized 33 AMR genes associated with resistance to beta-lactams, macrolides, glycopeptides, fluoroquinolones, and tetracyclines.

**Conclusion:**

Our findings highlight the importance of rapid, cost-effective molecular diagnostics in resource-limited settings. This approach facilitates early therapeutic decision- making by bypassing traditional culture-based methods. It also underscores the need for strengthened antibiotic stewardship and timely clinical intervention for critically ill patients in Bangladesh and similar contexts.

**Disclosures:**

All Authors: No reported disclosures

